# Dose–response effects of vitamin D supplementation on vitamin D status, glycosylated hemoglobin and total cholesterol in patients with diabetes mellitus: a systematic review and meta-analysis

**DOI:** 10.3389/fnut.2025.1663019

**Published:** 2025-11-06

**Authors:** Lu Cao, Nan Zhou, Dan Zhang, Peng Zhang, Ying Zhao

**Affiliations:** Department of Pharmacy, Shaanxi Provincial People’s Hospital, Xi’an, China

**Keywords:** diabetes mellitus, vitamin D levels, dose–response, glycosylated hemoglobin, total cholesterol, vitamin D supplementation

## Abstract

The impact of vitamin D levels on glycemic control and lipid metabolism in diabetic patients has received widespread attention. However, currently, there was no unified standard for vitamin D supplementation dosages, with significant variations among guideline recommendations. For instance, the 2022 ESPEN Guidelines recommended a daily supplementation of 1,500–5,000 International Units (IU) for patients at risk of vitamin D deficiency or who repeatedly experience vitamin D deficiency; however, guidelines from Italy suggested a daily supplement dose of 800–1,000 IU for patients with vitamin D deficiency. In this study, we searched the PubMed, Web of Science, Embase, Cochrane Library, CBM, CNKI, and Wanfang databases from their inception to 31 May 2024 for literature. The effects of different supplementation levels on vitamin D levels, glycosylated hemoglobin (HbA1c) levels and total cholesterol (TC) levels were analyzed using random-effects and fixed-effects models, respectively, and we applied the Modified Jadad Scale and the Newcastle-Ottawa Scale (NOS) score to evaluate the quality of the RCT studies and retrospective analyses, respectively. We included a total of seven papers involving 468 patients with a follow-up period of 3 to 6 months. The results of the study showed that vitamin D levels were significantly higher in the high-dose group than in the low-dose group at both 3 and 6 months of treatment [mean difference (MD) = −12.48, 95% confidence interval (CI): −15.25 to −9.72 and MD = −28.22, 95% CI (−40.92, −15.72), both *p* < 0.05], and the effect of prolonged treatment was more significant. HbA1c levels were significantly lower in the high-dose group than in the low-dose group [MD = 0.41, 95% CI (0.14, 0.67), *p* = 0.003], and TC levels were not significantly different between the two groups [MD = 1.84, 95% CI (−8.07, 0.67), *p* = 0.72]. Therefore, in patients with diabetes mellitus complicated by vitamin D deficiency, higher-dose supplementation (>4,000 IU/day) might have had potential advantages in increasing vitamin D levels and improving glycemic control. However, further studies were still needed to clarify the long-term safety and risk–benefit ratio of higher-dose supplementation.

## 1 Introduction

Vitamin D, as an essential nutrient for the human body, has a clear role in calcium and phosphorus balance, affecting bone health and neuromuscular function (1). The detection of serum 25-hydroxy vitamin D (25(OH)D) levels is widely recognized as the most reasonable indicator to reflect vitamin D status (2). With the deepening of research, its physiological functions has gone far beyond the traditional cognition. A study in 1988 first found that vitamin D receptors and vitamin D enzyme systems existed in most cells of the human body and could induce the transcription of hundreds of genes (3). A large number of subsequent studies further confirmed that serum 25(OH)D levels were closely related to the occurrence and development of various non-skeletal diseases. In the field of metabolic diseases, the association between vitamin D and diabetes mellitus is particularly significant (4). A meta-analysis by Mohammadi et al. (5) showed that vitamin D levels were negatively correlated with the risk of type 2 diabetes mellitus (T2DM) and pre-diabetes in adults, and each 10 ng/mL increase in serum 25(OH)D levels reduced the risk of T2DM by 12%. Further studies in recent years confirmed that vitamin D could not only reduce the incidence of diabetes mellitus in patients with impaired glucose tolerance, but also delay the progression of macrovascular and microvascular complications of diabetes (6).

Vitamin D deficiency is highly prevalent globally, affecting 60 to 80% of the population. This condition is associated with factors such as country of residence, race, ethnicity, and dietary habit (7). The situation is even more severe among diabetic patients, with up to 94.4% of them being vitamin D deficient, which is significantly higher than in the general population (8). Epidemiological studies have shown that low vitamin D levels are closely linked to insulin resistance, which is often accompanied by β-cell destruction and impaired glucose tolerance (9). In recent years, multiple studies have demonstrated associations between vitamin D levels and the development of diabetes mellitus, as well as the occurrence and progression of diabetic complications (5, 6, 10, 11). Data from the Third National Health and Nutrition Survey (2001–2014) showed that, among adult patients with diabetes mellitus, higher levels of vitamin D were associated with lower rates of cardiovascular and all-cause mortality (12).

However, different intakes and supplementation levels were recommended by different organizations. For example, the Guidelines for the Prevention and Treatment of Vitamin D Deficiency (2023 update in Polish) recommended vitamin D supplements ranging from 1,000 IU/d to 10,000 IU/d for patients with vitamin D deficiency (vitamin D level<20 ng/mL) (13). The 2022 ESPEN Guidelines for Micronutrients recommended that for patients at risk of or with recurrent vitamin D deficiency, the supplementation dosage should be 1,500–5,000 IU/d (10). The 2022 Italian Guidelines on the Definition, Diagnosis and Management of Vitamin D Deficiency recommended a daily supplementation of 800–1,000 IU for patients with vitamin D deficiency (14). The 2023 Expert Consensus on the Evaluation and Improvement of Vitamin D Nutritional Status, published by the Health Management Branch of the Chinese Society of Nutrition, recommended 400–800 IU/d for the prevention of vitamin D deficiency, and a daily intake of 2000 IU for patients with vitamin D deficiency (15). Additionally, both the Endocrine Society and the Chinese Society of Osteoporosis and Bone Mineral Research recommended 1,500–2,000 IU/d for high-risk adult patients with vitamin D deficiency; for adults with vitamin D deficiency, they recommended administration of vitamin D3 supplements at a dose of 6,000 IU/d or 50,000 IU/week for 8 weeks to achieve vitamin D levels above 30 ng/mL, followed by maintenance therapy with 1,500–2,000 IU/d (2, 16). In summary, the recommended dosage of vitamin D for patients with vitamin D deficiency was 400 IU/d to 10,000 IU/d.

We sorted out the evidence on which these guidelines recommendations were based and found that the clinical trial results on which most guidelines were based all came from healthy populations (2, 16–18). For example, the evidence on which the ESPEN guidelines were based came from healthy volunteers; its study showed that to make the average vitamin D levels of the normal weight, overweight and obese subgroups reach 40 ng/mL, 2,080 IU/d, 3,065 IU/d and 5,473 IU/d needed to be supplemented, respectively (19). But only the basis of the Polish guidelines included data from the diabetic population. The results of this study showed that among the population with serum 25(OH)D levels <20 ng/mL in the prediabetic stage, participants who supplemented 4,000 IU/d of vitamin D3, which made their serum 25(OH)D levels reach and maintain above 40 ng/mL, could gradually reduce the risk of prediabetic adults progressing to diabetes mellitus (20). Therefore, the supplementary dosages recommended by the existing guidelines could not well guide the population with diabetes mellitus complicated with vitamin D deficiency.

A double-blind, randomized, placebo-controlled clinical trial conducted by Niroomand et al. (21) showed that after 6 months of high-dose vitamin D supplementation (50,000 IU per week for the first 3 months and 50,000 IU per month for the next 3 months) in patients with prediabetes and vitamin D deficiency, their vitamin D levels increased significantly (36 ng/mL in the intervention group vs. 16 ng/mL in the control group, *p* < 0.001), and the Homeostatic Model Assessment of Insulin Resistance (HOMA-IR) score was significantly lower than that in the placebo group (2.6 vs. 3.1, *p* = 0.04). The proportion of patients progressing to diabetes mellitus in the intervention group was significantly lower than that in the control group (3% vs. 28%, *p* = 0.002). Ali et al. (22) compared oral supplementation of vitamin D at 4,000 IU/d and 50,000 IU/week in patients with T2DM. After 3 months, vitamin D levels in both groups increased significantly, with decreases in HbA1c levels, fasting insulin, and improvement in insulin resistance, but there was no significant difference between the two groups. Then, could a smaller dose meet the treatment needs? Does high dosage increase the risk of vitamin D overdose? The most frequent cause of vitamin D overdosing was exogenous, that is, excessive intakes. Due to the prolonged half-life of 25(OH)D, hypercalcemic-hypercalciuric syndrome might persist for weeks to months after treatment cessation, leading to significant morbidity and, in severe cases, extensive and irreversible soft tissue damage from mineral deposition. Vitamin D excess includes hypercalcemia, hypercalciuria, and mineral deposition in soft tissues (23). The safe upper limit of vitamin D supplements in normal-weight adults was recommended to be 4,000 IU/d (13, 15). Exploring an appropriate supplementary dosage to balance effectiveness and safety was an urgent problem to be solved.

Therefore, the objective of this study was to conduct a systematic review and meta-analysis of published randomized controlled trials (RCTs) and retrospective studies to compare the effects of low-dose (≤4,000 IU/d) versus high-dose (>4,000 IU/d) vitamin D3 on vitamin D levels, HbA1c, total cholesterol (TC) levels, and adverse events in adult patients with diabetes mellitus.

## 2 Materials and methods

### 2.1 Inclusion criteria

1. Study population: Participants were ≥18 years old, diagnosed with diabetes mellitus, and receiving vitamin D3 supplementation therapy;
2. Interventions/control measures: Administration of different doses of vitamin D3, with the low-dose group defined as ≤4,000 IU/d and the high-dose group as >4,000 IU/d. The route of administration and treatment duration were not restricted;
3. Outcome measures: Primary outcomes included serum 25(OH)D levels, HbA1c levels, and serum TC levels; and
4. Study design: Eligible studies were published randomized controlled trials (RCTs) or retrospective studies.

### 2.2 Exclusion criteria

(1) Vitamin D has no biological activity, and need to be hydroxylated in two steps by the liver and kidney to form 1,25-dihydroxyl vitamin D (1,25(OH)_2_D), that is, active vitamin D, to have biological activity and function. Active vitamin D does not require metabolism by the liver or kidneys, and can directly exert physiological functions. Therefore, the function of vitamin D was affected by liver and kidney function, but active vitamin D was not affected by this. Vitamin D had a slow onset in the body, a long action time, a fast action of active vitamin D, a short action time, and a high risk of hypercalcemia of active vitamin D (2). In order to reduce the impact of confounding factors, this study focused on vitamin D3 supplementation and excluded articles grouped as active vitamin D; (2) even in overweight or obese adults, the upper limit of vitamin D3 intake recommended by the guidelines is 10,000 IU/d (13), so we excluded articles with group doses above 10,000 IU/d; (3) outcome metrics that were not elucidated in relation to the dose ranges defined in this article; (4) outcome metrics that were not included in the study; (5) repetitively published literature; (6) literature based solely on case reports; (7) literature not written in Chinese or English; and (8) literature for which the full text could not be obtained, even after contacting the authors.

### 2.3 Literature search strategy

Computerized searches were conducted in the following databases: PubMed, Web of Science, Embase, the Cochrane Library, the China Biomedical Literature Database (CBM), the China National Knowledge Infrastructure (CNKI) and Wanfang. The search period covered the time from the date of database construction to May 2024. The English search terms were as follows: (“diabetes mellitus” OR “diet, diabetic” OR “prediabetic state” OR “glucose intolerance” OR “diabetes”) AND (“vitamin D” OR “receptors, calcitriol”) AND (“dosage” OR “dose”). The Chinese search terms were as follows: diabetes, vitamin D3, dose. A combination of subject and free word searches was used, adapted for each specific database. The references of the included studies were also searched to obtain additional relevant information.

Articles retrieved through the search strategy were first deduplicated using EndNote X9. Subsequently, two researchers (LC and YZ) independently conducted screening and evaluation, which included assessing the titles, abstracts, main texts, and supplementary materials. Research data were extracted from the main texts. When there were differences of opinion between the two researchers, a third reviewer was involved to evaluate the relevance of the disputed articles and their compliance with the inclusion and exclusion criteria. We calculated the level of agreement between the researchers’ opinions using Cohen’s Kappa statistic.

### 2.4 Data screening and extraction

Two researchers independently reviewed the literature and extracted information, and then cross-checked the results. If there is a disagreement between the two parties, they will first conduct negotiations. The two researchers will conduct full discussions on the points of disagreement, check the original literature information, and clarify the basis for their respective judgments. If an agreement is reached through discussion, the final data extraction will be carried out. If there is still a disagreement after the initial negotiation, a third researcher who did not participate in the initial review (the researcher should have professional background in the relevant field and be unaware of the judgments of the first two researchers) needs to be invited. The third researcher will independently review the controversial literature and provide the final judgment and reasons. The three researchers will discuss the third party’s review opinions together, recheck the points of disagreement with reference to the original literature information, and finally determine the extracted data based on the majority opinion. The extracted information comprised the following:

1. Basic information of the literature (first author, publication date, country and study design);
2. Clinical characteristics of the subjects (age, number of cases, baseline vitamin D level);
3. Interventions and controls (type of vitamin D3 supplementation, route, dosage and duration of treatment);
4. Outcome [serum 25(OH)D levels, HbA1c, TC]; and
5. Indicators related to the quality evaluation of the literature.

### 2.5 Evaluation of the quality of included studies

RCT studies were evaluated using the modified Jadad scale (24), with the evaluation items including the generation of random sequences, randomization concealment, blinding, withdrawal and loss of visits, and the combined scores of the aforementioned items. Studies receiving 1–3 points were classified as low-quality, while those receiving 4–7 points were classified as high-quality. The Newcastle-Ottawa Scale (NOS) was utilized to evaluate retrospective studies. The NOS encompasses the following criteria: the representativeness of the study population, the comparability of the study populations, the sufficiency of the follow-up period, and the adequacy of the follow-up evaluation. Additionally, it addresses issues of loss to follow-up and dropout. The NOS score was derived by combining these items and the literature, yielding a range from 5 to 10. Studies with a higher NOS score exhibit reduced bias and were deemed eligible for inclusion in the meta-analysis. It was noteworthy that the NOS score is inversely proportional to bias, with higher scores indicating reduced bias (25).

### 2.6 Statistical analysis

The statistical analysis was conducted using Rev. Man 5.3.3 software. In this study, the outcome indicators were all continuous variables; therefore, they were expressed by using the mean difference (MD) and its 95% confidence interval (CI) to combine. The Cochrane Q-test was utilized to assess the heterogeneity of the results obtained from the included studies. When *p* > 0.1 and *I*^2^ ≤ 50%, it was indicative of the absence of a statistically significant difference in heterogeneity between the studies, thereby permitting the implementation of a fixed-effects model for the combined analyses. Conversely, when *p* < 0.1 and *I*^2^ > 50%, a random-effects model was deemed appropriate for the combined analyses. The publication bias test was completed for the outcome indicators to generate a funnel plot and to detect whether there was publication bias in the literature included in the meta-analysis by qualitatively determining whether the distribution of scatter points was symmetrical or not. The observed difference was found to be statistically significant at *p* < 0.05.

## 3 Results

### 3.1 Literature search results

**Figure 1:**
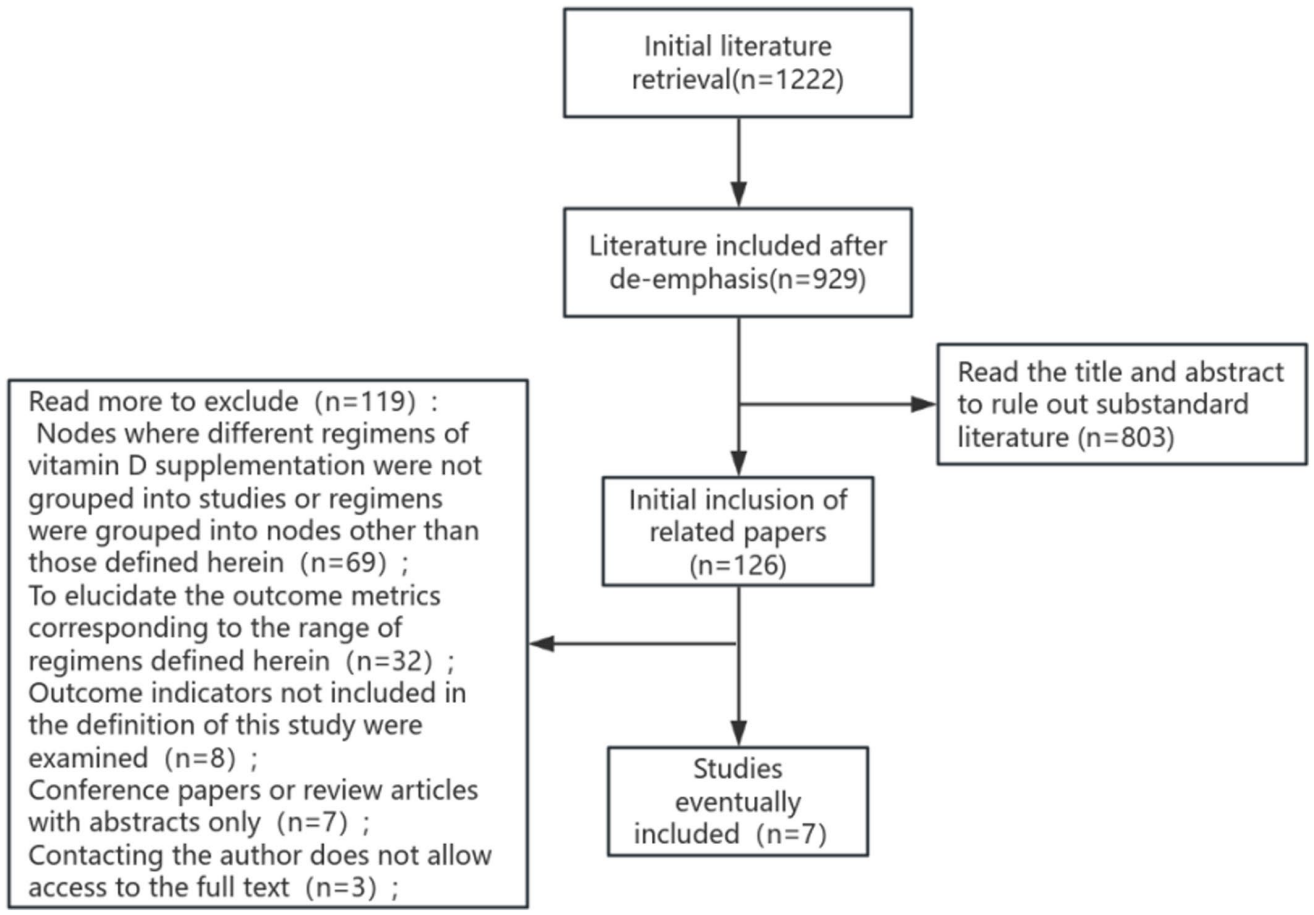
Literature screening flow chart.

The proposed search terms were entered into the database, and a total of 1,222 papers were detected, including 469 papers in English and 753 papers in Chinese. Following a rigorous selection process, seven literatures were ultimately selected for systematic evaluation and meta-analysis. The process of literature screening is illustrated in Figure 1.

### 3.2 Basic characteristics of the included studies

A total of 468 patients were enrolled in seven studies (22, 26–31), of whom 261 were allocated to the low-dose group (≤4,000 IU/d) and 207 to the high-dose group (>4,000 IU/d). The experimental and control groups were meticulously designed by each study, with consideration given to the fundamental characteristics of the patients and the initial conditions of the two groups, ensuring their comparability. The basic characteristics of the included studies were displayed in Table 1.

**Table 1:** Basic characteristics of the included studies.

First author, data	Country	Study type	Population	Age (years)	Baseline vitamin D level (ng/ml)	Drugs	Low-dose group: ≤4,000 IU/d		High-dose group:>4,000 IU/d		Course	Outcome measures	Modified Jadad or NOS
							Regimen	Participants (*n*)	Regimen	Participants (*n*)			
Ali et al. (22)	Egypt	RCT	T2DM	≥18	<20	**Oral/injectable vitamin D3**	4,000 IU/d	20	50,000 IU/week	20	3 months	HbA1c; serum 25(OH)D levels	5
							300,000 IU/3 months	20					
Exebio et al. (27)	United States	RCT	T2DM and vitamin D insufficiency	30–70	<30	**Oral vitamin D3**	4,000 IU/d	50	6,000 IU/d	25	6 months	TC; serum 25(OH)D levels	5
Felício et al. (28)	United States	Interventional study	T1DM	18–50	30–60 < 30	Oral Vitamin D	4,000 IU/d	6	10,000 IU/d	16	12 weeks	serum 25(OH)D levels	9
Habiba et al. (26)	Egypt	RCT	T2 DM	30–65	<20	Oral vitamin D3	4,000 IU/d	20	50,000 IU/week	20	3 months	TC; serum 25(OH)D levels	6
							300,000 IU/3 months	20					
Karonova et al. (29)	Russia	RCT	T2DM and diabetic neuropathy	18–65 years	<30 (accounting for 78%)	Oral vitamin D3	5,000 IU/week	31	40,000 IU/week	31	24 weeks	TC; serum 25(OH)D levels; HbA1c	6
Lei et al. (31)	China	RCT	Diabetes mellitus and periodontitis	≥18 years	<30	Oral vitamin D3	25,000 IU/week	30	50,000 IU/week	30	6 months	serum 25(OH)D levels; HbA1c	7
Penckofer et al. (30)	United States	RCT	T2DM with depressive symptoms	≥21 years	<32	Oral vitamin D3	5,000 IU/week	64	50,000 IU/week	65	6 months	serum 25(OH)D levels	6

### 3.3 Evaluation of the methodological quality of the included literature

In the present study, a total of seven papers were included for analysis. The kappa coefficient was 0.74 (moderate). Of these, six were found to be randomized controlled trials (RCTs), and the Modified Jadad Scale assessment revealed that all of these studies were of a high quality (scores ranging from 4 to 7). The remaining paper was a retrospective study, and this study received a NOS score of 9. The overall methodological quality was deemed to be fair, and the specific scores were outlined in Table 1.

### 3.4 Results of meta-analysis

#### 3.4.1 Effects of different vitamin D supplementation doses on serum 25(OH)D levels

**Figure 2:**
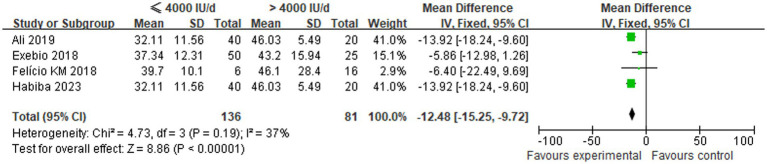
Comparison of serum 25(OH)D levels between the two groups after 3 months of different-dose vitamin D supplementation.

A total of four studies compared the changes in serum 25(OH)D levels between low-dose and high-dose vitamin D supplementation groups after 3 months. Statistical heterogeneity was observed among the included studies (*p* = 0.19, *I*^2^ = 37%), necessitating the use of a fixed-effects model for analysis. The meta-analysis demonstrated that the mean vitamin D levels in the ≤4,000 IU/d supplementation group were significantly lower than those in the >4,000 IU/d group. This finding was accompanied by a statistically significant difference between the two groups [MD: −12.48, 95% CI (−15.25, −9.72), *p* < 0.00001], as illustrated in Figure 2.

**Figure 3:**
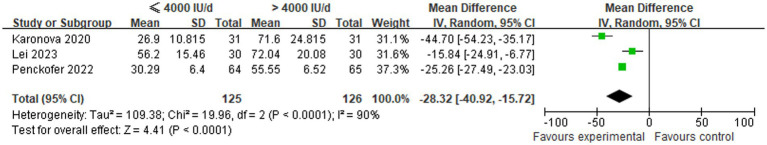
Comparison of serum 25(OH)D levels between groups following 6 months of different-dose vitamin D supplementation.

A total of three studies were conducted to compare the changes in serum 25(OH)D levels between low-dose and high-dose vitamin D supplementation groups after a period of 6 months. Statistical heterogeneity was identified among the included studies (*p* < 0.00001, *I*^2^ = 90%), thus necessitating the implementation of a random-effects model for the analysis. The results of the meta-analysis demonstrated that the mean serum 25(OH)D levels in the ≤4,000 IU/d supplementation group were significantly lower than those in the >4,000 IU/d group, with a statistically significant intergroup difference [MD: −28.32, 95% CI (−40.92, −15.72), *p* < 0.001], as illustrated in Figure 3.

#### 3.4.2 Effects of different doses of vitamin D supplementation on HbA1c

**Figure 4:**
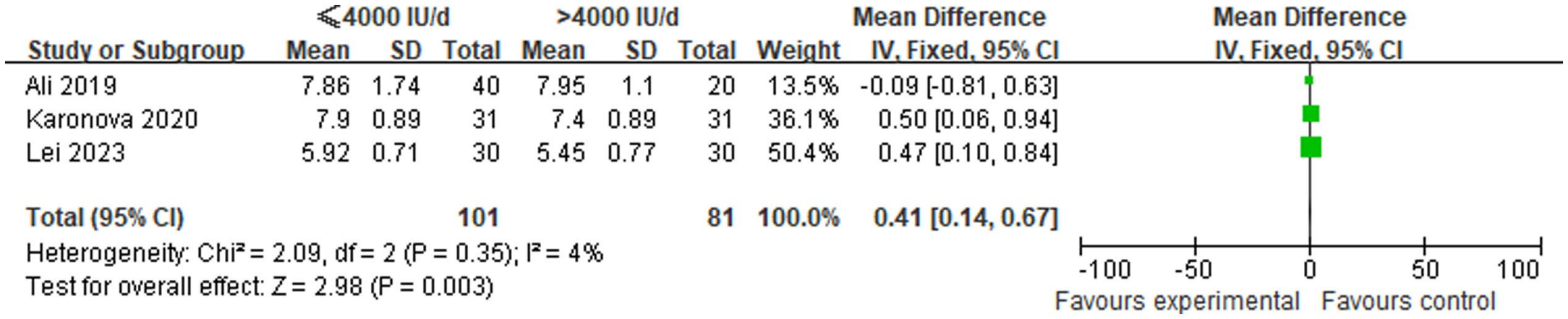
Comparison of HbA1c levels among groups undergoing different doses of vitamin D supplementation.

A comparison was made of changes in HbA1c levels between low-dose (≤4,000 IU/d) and high-dose (>4,000 IU/d) vitamin D supplementation groups in three studies. In view of the absence of statistical heterogeneity among the included studies (*p* = 0.35, *I*^2^ = 4%), a fixed-effect model was employed for the meta-analytic synthesis. The pooled analysis demonstrated a statistically significant increase in HbA1c levels in the ≤4,000 IU/d group compared to the >4,000 IU/d group, with WMD of 0.41 [95% CI (0.14, 0.67), *p* = 0.003], as illustrated in Figure 4.

#### 3.4.3 Effects of different vitamin D supplementation doses on TC levels

**Figure 5:**
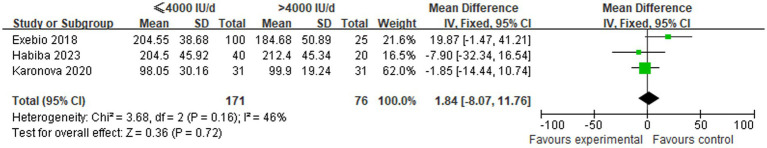
Comparison of TC levels between the two groups following supplementation with varying doses of vitamin D3.

Three studies were included in order to make a comparison between the changes in TC levels between the low-dose and high-dose vitamin D3 supplementation groups. In view of the absence of statistical heterogeneity across the studies (*p* = 0.16, *I*^2^ = 46%), a fixed-effect model was employed for meta-analytic integration. The pooled analysis indicated that the ≤4,000 IU/d group exhibited marginally higher TC levels in comparison to the >4,000 IU/d group. However, the observed difference between these groups did not attain statistical significance [MD: 1.84, 95% CI (−8.07, 11.76), *p* = 0.72], as shown in Figure 5.

#### 3.4.4 Adverse events

Six studies did not report any vitamin D-related adverse events. One study reported that two subjects experienced hypercalcemia, with one case in the high-dose group and one case in the low-dose group.

### 3.5 Publication bias

**Figure 6:**
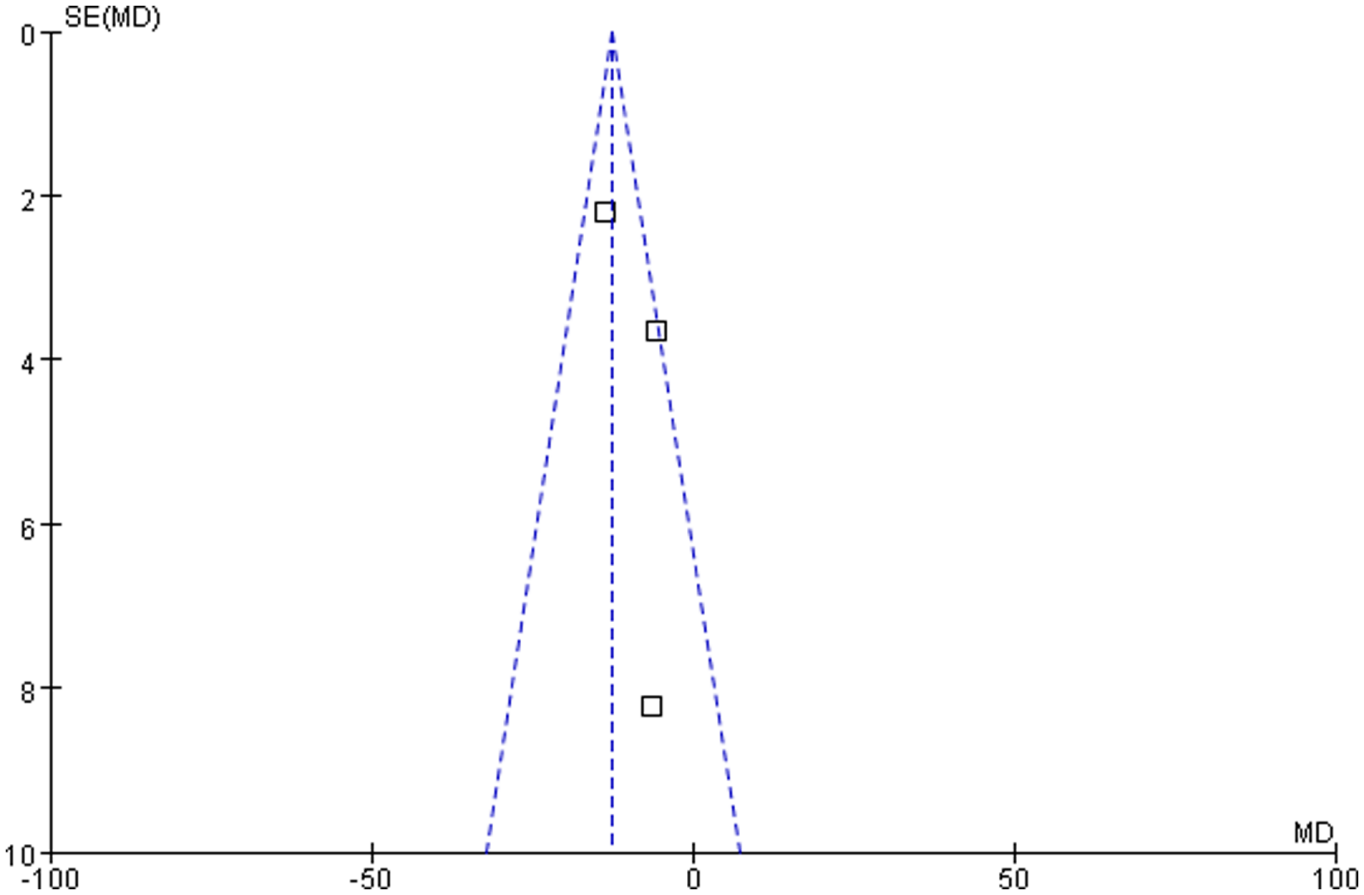
Funnel plot of vitamin D level comparison between groups following 3 months of different-dose vitamin D3 supplementation.

As demonstrated in Figure 6, the funnel plot for the outcome of serum 25(OH)D levels following 3 months of different-dose vitamin D supplementation exhibited a relatively symmetrical scatter distribution. This finding suggested the absence of any discernible publication bias in this meta-analysis.

## 4 Discussion

In order to interpret the effects of different doses of vitamin D in patients with diabetes mellitus, it was necessary to consider the intervention types and research design analyses of the included studies. In this study, the focus was exclusively on the comparative analysis of vitamin D3 supplements, devoid of any concomitant micronutrient combinations. This methodological approach was adopted to ascertain the precise effects of vitamin D within a predetermined dose range.

The Endocrine Society of the United States holds that there is no clinical research evidence to support the establishment of different serum 25(OH)D thresholds based on the benefits of specific outcomes in the studied populations (32). However, guidelines for the diagnosis and treatment of osteoporosis recommended that serum 25(OH)D levels should be maintained at 30 ng/mL to preserve skeletal health (33). Currently, for diabetes mellitus patients, there was no guidelines clearly specifying the threshold at which serum 25(OH)D levels should be maintained to obtain metabolic benefits. A study by Chen et al. (34) showed that compared with people with serum 25(OH)D levels of at least 30 ng/mL, those with serum 25(OH)D levels lower than 10 ng/mL had a lower risk of diabetic microvascular complications (including diabetic retinopathy, diabetic nephropathy, and diabetic neuropathy). Xu et al. (35) conducted a study on 1,202 type 2 diabetic patients with nephropathy and found that higher serum 25(OH)D levels were significantly associated with reductions in all-cause mortality, non-accidental mortality, and malignant tumor mortality, that is, the higher the serum 25(OH)D levels, the lower the risk of death. The results of a randomized controlled trial of vitamin D supplementation in pre-diabetic patients showed that compared with participants who maintained serum 25(OH)D levels of 20–30 ng/mL, the risk ratios of developing diabetes in participants treated with vitamin D who maintained serum 25(OH)D levels of 40–50 ng/mL and ≥50 ng/mL were 0.48 (0.29–0.80) and 0.29 (0.17–0.50), respectively (20). The results of this study showed that after 3 months and 6 months of treatment, the vitamin D level in the high-dose group was significantly higher than that in the low-dose group (the MD were −13.93 and −22.06, respectively, both *p* < 0.05). However, the average concentrations in both groups reached a non-deficient state of ≥30 ng/mL, suggesting that the low dose might have been sufficient to meet the needs of bone health. It is worth noting that among the studies we included, except for the study by Lei et al. (31), serum 25(OH)D levels in the low-dose group were <40 ng/mL at 3 months and 6 months, while those in the high-dose group were >40 ng/mL. Previous studies have demonstrated that the attainment of 40–60 ng/mL is necessary for vitamin D to demonstrate substantial extra-skeletal effects, including the enhancement of insulin resistance (36). Therefore, to obtain metabolic benefits, it might have been necessary to administer high-dose vitamin D supplementation. However, there were many factors affecting vitamin D levels, including age, skin color, season, geographical latitude, altitude, dressing habits, sun exposure time, sun protection measures, dietary habits, air pollution, obesity, and drugs affecting vitamin D metabolism (2). Therefore, the supplementary dose of vitamin D should be comprehensively considered in combination with individual factors.

HbA1c was the primary measure for assessing glycemic status in both clinical practice and trials, reflecting average blood glucose levels over approximately 2–3 months and demonstrating a close association with diabetes complications (37). Vitamin D influenced insulin synthesis and secretion through multiple pathways: its active metabolite, 1,25-dihydroxyvitamin D3 (1,25(OH)2D), bound to the vitamin D receptor (VDR), not only enhanced glucose transport but also directly stimulated insulin release from pancreatic β-cells. Additionally, vitamin D potentially optimized glucose metabolism by regulating intracellular calcium homeostasis, thereby affecting blood glucose levels (38). A previous meta-analysis reported that vitamin D supplementation significantly reduced HbA1c compared with placebo [WMD: −0.30% (95% CI: −0.43 to −0.18), *p* = 0.000] (39). However, this study did not compare different supplementation doses, and the included studies used a wide dose range (400–11,200 IU/d). Another meta-analysis stratified doses into ≤2,000 IU/d and >2,000 IU/d groups but found no significant HbA1c reduction in either [WMD: −0.21, 95% CI (−0.53, 0.11), *p* = 0.189; and WMD: 0.05, 95% CI (−0.41, 0.51), *p* = 0.832] (40). The dose range in this study was similarly broad (20–11,200 IU/d), particularly in the high-dose group, where the minimum and maximum doses differed by 9,200 IU/d. This substantial heterogeneity likely contributed to the discrepant results between the two meta-analyses. Neither of these two studies directly compared the effects of high-dose versus low-dose vitamin D supplementation on HbA1c. Our study revealed that patients receiving >4,000 IU/d of vitamin D had significantly lower HbA1c levels than those receiving ≤4,000 IU/d [MD = −0.41, 95%CI (−0.67, −0.14), *p* = 0.003]. Although this difference was slightly below the conventional clinical significance threshold (0.5%), it suggested that higher doses of vitamin D may have an HbA1c-lowering effect. Importantly, diabetes mellitus management required a comprehensive approach encompassing multiple targets (e.g., fasting glucose, blood pressure, lipids). Further research was needed to evaluate the impact of vitamin D doses on these additional metabolic parameters. Moreover, individual factors (e.g., baseline vitamin D status, comorbidities) might influence treatment responses, necessitating stricter dose-comparison studies and personalized analyses in future investigations.

In the field of lipid metabolism, there was a degree of contention surrounding the relationship between vitamin D levels and TC. A cross-sectional study involving 278 young adults showed a negative correlation between vitamin D levels and TC (*ρ* = −0.316, *p* = 0.014) (41), while a cross-sectional analysis by Gholamzad et al. (42) in 15,600 healthy participants found no significant association (*p* > 0.05). In a meta-analysis on the effects of vitamin D supplementation on blood lipids in T2DM, MacGirlley et al. (43)also indicated that there was no statistically significant difference in TC levels between the vitamin D supplementation and placebo groups [SMD = −0.16, 95% CI (−0.57, 0.24), *p* = 0.43]. In contrast, an umbrella study by Radkhah et al. (44) incorporating 25 meta-analyses demonstrated that vitamin D supplementation significantly reduced TC levels [ES: −0.17, 95% CI (−0.23, −0.11), *p* ≤ 0.001]. The results of this study showed that TC levels were slightly higher in the ≤4,000 IU/d vitamin D3 supplementation group than in the >4,000 IU/d group, though this difference was not statistically significant [MD = 1.84, 95% CI (−8.07, 11.76), *p* = 0.72]. The discrepancy between this finding and the conclusion of Radkhah et al. (44) might have been related to the wide range of vitamin D doses (20–5,000 IU/d) included in their study, suggesting that dose heterogeneity could have been a key factor contributing to the conflicting conclusions across existing studies.

It was worth noting that in the studies included in this analysis, the baseline serum 25(OH)D levels of most subjects were insufficient or deficient. Among them, the baseline serum 25(OH)D levels in two studies (22, 26) were lower than 20 ng/mL; the baseline serum 25(OH)D levels of subjects in three studies (27, 30, 31) were lower than 32 ng/mL; and in another study (29), 78% of the subjects had vitamin D deficiency or insufficiency. Studies indicated that for individuals with sufficient vitamin D (serum 25(OH)D ≥ 30 ng/mL), vitamin D supplementation had little preventive effect on major health outcomes such as cardiovascular events, cancer incidence, progression of T2DM, fracture risk, or all-cause mortality. In contrast, in populations with severe deficiency (serum 25(OH)D < 20 ng/mL), vitamin D supplementation showed pleiotropic benefits beyond bone health, including delaying the progression from prediabetes to diabetes, enhancing respiratory immunity, and possibly reducing tumor-related mortality and all-cause mortality (45–47). Jorde et al. (48) showed that for subjects without vitamin D deficiency, vitamin D supplementation was unlikely to prevent the progression from prediabetes to diabetes. In another study, an analysis of patients with severe vitamin D deficiency found that vitamin D supplementation could significantly reduce the risk of progression from prediabetes to diabetes (20). Therefore, supplementation with higher doses of vitamin D was not applicable to all patients and needed to be judged based on serum 25(OH)D levels.

Existing evidence suggested that body mass index (BMI) might have a mediating or regulating effect on vitamin D metabolism (49). Multiple studies confirmed that BMI was negatively correlated with serum 25(OH)D levels, and the increase in serum 25(OH)D levels after vitamin D supplementation in people with high BMI was relatively limited (49–51). This phenomenon might be related to the accumulation of vitamin D in adipose tissue, or it might result from the increased activity of 1-*α* hydroxylase caused by elevated parathyroid hormone levels in obese patients, thereby accelerating the conversion of 25(OH)D to the active form 1,25-dihydroxyvitamin D (52, 53). It was worth noting that some studies reported that high BMI was not only associated with a decrease in serum 25(OH)D levels but might also independently affect the increase in glycated hemoglobin (HbA1c) and dyslipidemia (54, 55). Among the studies included in this analysis, the subjects’ BMI in three studies (22, 28, 31) ranged from 24 to 29.9 kg/m^2^, and three studies (27, 29, 30) included subjects with BMI ≥ 30 kg/m^2^. However, only one study (28) evaluated the changes in BMI before and after the intervention, so subgroup analysis on the impact of BMI on efficacy could not be conducted. Based on this, future studies should design randomized controlled trials with different doses (especially >4,000 IU/d) and strictly control metabolic factors such as BMI to clarify the impact of vitamin D on metabolic indicators.

Studies found that vitamin D-related genetic polymorphisms could regulate the intervention effect by affecting the vitamin D metabolic pathway. As a member of the nuclear receptor superfamily, the vitamin D receptor (VDR) formed a VDR-RXR heterodimer by binding to 1α,25-dihydroxyvitamin D3, thereby regulating the transcription of genes related to calcium and phosphorus metabolism, cell proliferation, and immune regulation (56). Polymorphisms in the VDR gene might change the activity of VDR, leading to differences in individual responses to vitamin D supplements (57). Usategui-Martín et al. (56) found that the Tt + tt genotype of the VDR gene TaqI (rs731236) polymorphism and the FF genotype of FokI (rs10735810) were associated with a more significant response to vitamin D supplementation. This might be because the TaqI variation enhanced mRNA stability, while the FF type of FokI promoted the translation of more active proteins (57, 58). In addition, gene–gene interactions in the vitamin D metabolic pathway were also confirmed to affect clinical outcomes. For example, the interaction between the RXRG gene (rs2134095) polymorphism and the GC gene (rs7041) polymorphism could regulate LDL-c levels (59), and the AA genotype of the GC gene (rs4588) polymorphism was associated with poor blood glucose control (60). Although these genetic variations might partially explain the heterogeneity between studies, due to the fact that most included studies did not provide the genotype data of the subjects, it was currently impossible to systematically evaluate the impact of genetic factors on the outcome indicators of this study. Therefore, the impact of vitamin D-related gene polymorphisms on clinical outcomes still needed to be verified through well-designed genetic studies.

Vitamin D intoxication is a clinical condition characterized by excessive vitamin D, with main clinical manifestations including hypercalcemia, hypercalciuria, and mineral deposition in soft tissues (23). Excessive intake of vitamin D is one of the main pathogenic factors (61). For example, after the UK Department of Health recommended reducing vitamin D intake, the number of cases of infantile hypercalcemia decreased significantly (62). It was previously believed that 4,000 IU/d was the upper limit for vitamin D supplementation, but recent studies have put forward different viewpoints (23). A 4-year study found that monthly high-dose vitamin D3 supplementation had no impact on the adverse events reported by the subjects (63); another 3-year study showed that the safety of daily supplementation with 400 IU, 4,000 IU, and 10,000 IU was similar, although mild hypercalcemia occurred more frequently at higher doses (64). Currently, the safe upper limit of serum 25(OH)D level to avoid hypercalcemia has not been clearly defined, but most studies have shown that attention is only needed when the serum 25(OH)D level is >150 ng/mL. Therefore, setting 100 ng/mL as the tolerable upper intake level can provide a safety margin for reducing the risk of hypercalcemia (39). Guidelines suggested that if serum 25(OH)D levels>100 ng/mL, supplementation should be stopped immediately and monitored until the level is <50 ng/mL; if it is 50–100 ng/mL, the treatment plan (such as dose, compliance, and preparation type) needs to be evaluated and adjusted; if it is 30–50 ng/mL, the original plan can be maintained; if it is ≤30 ng/mL, the rationality of treatment needs to be re-evaluated and management optimized (13). In addition, high-dose vitamin D supplementation may have an impact on bone health, fall risk, kidney stones, etc. For example, in a randomized controlled trial, 311 healthy, vitamin D-sufficient, non-osteoporotic subjects were given 400, 4,000, or 10,000 IU/d of vitamin D, respectively, (65). At the end of 3 years, the changes in volumetric bone mineral density (BMD) at the distal radius in the 400, 4,000, and 10,000 IU/d groups were −1.2, −2.4%, and −3.5%, respectively, and the values in the latter two groups were significantly lower than those in the 400 IU group. At the distal tibia, only the volumetric BMD in the 10,000 IU group was lower than that in the 400 IU group. There was no difference in areal BMD at the total hip. The risk of adverse events might depend not only on the dose but also on the treatment effect, treatment regimen, and possibly age, sex, and vitamin D status (23, 65).

Only one of the studies included in this analysis reported two cases of hypercalcemia in the subjects, so we could not determine whether there was a difference in hypercalcemia between the high-dose group and the low-dose group. In addition, there were no other vitamin D-related adverse events in this study, which might be because most of the subjects included in our study were vitamin D deficient or insufficient. Therefore, we believed that short-term high-dose vitamin D supplementation for patients with vitamin D insufficiency or deficiency might be safe. However, due to the short follow-up period included in our study, we could not determine whether long-term use of high-dose vitamin D supplements was safe.

This study had several strengths. First, to our knowledge, it was the first meta-analysis to explore the dose–response effects in diabetic patients, filling a gap in this field. Unlike studies that simply compared supplementation versus non-supplementation or generalized dose groups, this study directly compared the efficacy differences between high and low doses, which could directly provide references for clinical dose selection. In addition, this study focused on the specific population of diabetic patients, avoiding the extrapolation bias of research results from other populations. The selected outcome indicators (25(OH)D level, HbA1c, TC) were all core indicators for diabetes management, which were in line with the modern concept of comprehensive diabetes management. Furthermore, this study ensured the rigor of the process and the reliability of the results through comprehensive literature retrieval, independent screening by two reviewers, and strict quality evaluation. Moreover, most of the included studies were randomized controlled trials with high quality scores, which further enhanced the persuasiveness of the conclusions. Finally, vitamin D deficiency is very common in diabetic patients, and the rational selection of vitamin D supplement doses is an urgent problem to be solved in clinical practice. However, there was no relevant meta-analysis, which highlighted the special significance of this study.

This study also had some limitations: (i) due to the small number of included studies, this might have affected the homogeneity, similarity, and consistency required for the meta-analysis; (ii) BMI might be one of the factors affecting 25(OH)D levels, thereby affecting other outcome indicators. However, among the 7 included literatures in this study, only one reported the change in BMI before and after intervention, so this study could not conduct a subgroup analysis of BMI or determine the impact of BMI on outcome indicators; (iii) the included studies lacked data on vitamin D gene polymorphism, making it impossible to judge the impact of gene polymorphism on outcome indicators; (iv) the sample size was small, most of which were small-scale exploratory studies, and the included studies had a short duration, making it difficult to identify potential effects or safety issues. Therefore, the conclusions of this study could provide certain reference value for clinical practice, but further verification by randomized controlled trials with larger samples and longer follow-up periods is still needed.

## 5 Conclusion

The results of this study showed that in patients with type 2 diabetes mellitus complicated by vitamin D deficiency, the high-dose vitamin D supplementation regimen of >4,000 IU/d could more significantly correct the vitamin D deficiency state and improve glycemic control compared with the low-dose regimen of ≤4,000 IU/d. This finding provided new clinical evidence for the role of high-dose vitamin D supplementation in blood glucose regulation in patients with type 2 diabetes mellitus. However, in view of the potential risks that might be brought by high-dose treatment, safety indicators such as serum calcium, serum phosphorus, and bone mineral density should be closely monitored in clinical practice to evaluate its long-term safety. When formulating individualized treatment plans, it was necessary to comprehensively consider the patients’ baseline vitamin D levels, metabolic characteristics, and complication risks to optimize the balance between efficacy and safety.

## Data Availability

The datasets presented in this study can be found in online repositories. The names of the repository/repositories and accession number(s) can be found in the article/supplementary material.
